# Tending to innovate in Swedish primary health care: a qualitative study

**DOI:** 10.1186/s12913-019-3874-y

**Published:** 2019-01-18

**Authors:** Gunilla Avby, Sofia Kjellström, Monica Andersson Bäck

**Affiliations:** 1 The Jönköping Academy for Improvement of Health and Welfare, Jönköping University, Box 1026, 551 11 Jönköping, Sweden; 2 Department of Social Work, University of Gothenburg, Box 720, 405 30 Göteborg, Sweden

**Keywords:** Primary care, Health care reform, Practice features, Innovation, Leadership, Culture and climate for innovation

## Abstract

**Background:**

Policymakers in many countries are involved in system reforms that aim to strengthen the primary care sector. Sweden is no exception. Evidence suggests that targeted financial micro-incentives can stimulate change in certain areas of care, but they do not result in more radical change, such as innovation. The study was performed in relation to the introduction of a national health care reform, and conducted in Jönköping County Council, as the region’s handling of health care reforms has attracted significant national and international interest. This study employed success case method to explore what enables primary care innovations.

**Methods:**

Five Primary Health Care Centres (PHCCs) were purposively selected to ensure inclusion of a variety of aspects, such as size, location, ownership and regional success criteria. 48 in-depth interviews with managers and staff at the recruited PHCCs were analysed using content analyses. The COREQ checklist for qualitative studies was used to assure quality standards.

**Results:**

This study identified three types of innovations, which break with previous ways of organizing work at these PHCCs: (1) service innovation; (2) process innovation; and (3) organizational innovation. A learning-oriented culture and climate, comprising entrepreneurial leadership, cross-boundary collaboration, visible and understandable performance measurements and ability to adapt to external pressure were shown to be advantageous for innovativeness.

**Conclusions:**

This qualitative study highlights critical features in practice that support primary care innovation. Managers need to consistently transform and integrate a policy “push” with professionals’ understanding and values to better support primary care innovation. Ultimately, the key to innovation is the professionals’ engagement in the work, that is, their willingness, capability and opportunity to innovate.

**Electronic supplementary material:**

The online version of this article (10.1186/s12913-019-3874-y) contains supplementary material, which is available to authorized users.

## Background

### Primary care in the overall Swedish health care system

Primary care is described as a sector highly influenced by political decisions and defined by its imperative role in the overall health care system [1, 2]. Policymakers in many countries are involved in system reforms that aim to strengthen the primary care sector [3]. The Nordic countries are no exception. The Nordic region in northern Europe consists of Finland, Denmark, Norway and Iceland, as well as Sweden. It has been suggested that there is a certain “Nordic Model” for health care systems, as an integral part of the Nordic welfare model [4]. The system is governed by the population structure and rules of law, an important feature being that there are no new patients or customers, rather the value-creating process must be maximised within the given framework and with responsibility for high quality and safety [1, 5]. We know that the Nordic health care systems share a number of common core aspirations, such as equity and participation, but also that there is considerable variation at the structural and policy level among the Nordic countries in the way strategies are designed, conceived and implemented [4]. However, to handle the social challenges of today, a common structural and policy aspect is that Nordic health care systems are gradually putting more weight on financial imperatives and looking for more diversity in the provision of services. The practice of looking to the private sector for managerial ideas is often a basic component of these new systems, because private undertakings are considered to provide reliable tools for the necessary transformation in values, work, and organization [6, 7]. Evidence suggests that targeted financial micro-incentives can stimulate change in certain areas of care, but they do not result in more profound service transformations [2].

To understand Swedish primary care, two things are important. First, primary care has traditionally been a public monopoly based on a value ground of equity, with a shift of power since the 1980s resulting in a decentralized health care system [8, 9]. Second, Swedish primary health care centres (PHCCs) employ a multidisciplinary work force, typically 4–10 physicians specialized in general practice working together with several other professionals, such as nurses, physical and occupational therapists, social workers, psychologists, nurses, nurse assistants, and administrators [8]. In a global comparison, the multidisciplinary characteristics of health care centres tend to be quite unusual, although similar systems exist in Finland, the UK, and the Netherlands [9].

In recent years, the growing demands for care from a rapidly aging population, the recognition of high levels of lifestyle-related health problems, politicians’ devotion to reducing tax levels (precluding additional public funds for health care), and difficulties attracting qualified personnel have highlighted the need for new solutions in the overall Swedish health system. A major concern has been the ability to meet the requirements for improved continuity of care for patients, especially elderly patients. Primary care has been identified as having an imperative role to meet these demands, and the PHCCs have been appointed in the role of “gatekeepers” to unburden hard-pressed hospitals, support equal health care and enable necessary health services transformation [1]. Also, innovation has been singled out to help us transform and deliver a national health service for the twenty-first century [10–14]. In 2007, the National Choice of Care Reform to improve performance and strengthen the role of primary care was initiated, providing choice, new reimbursement models, and an opening for private and non-profit actors to compete. The guidelines supporting the reform express the idea that an organizational change of primary care will enable improved practice outcome, such as better accessibility and choice of care for patients, higher quality in services, and practice transformation.

However, health care reforms are thought to cause problems, such as increasing work load, undermining professional autonomy, having negative effects on staff morale and fortifying management values and priorities for controlling employees [6, 15, 16]. We have found two literature reviews on the reform; one claiming that few innovations have resulted from the reform [8] and one stressing that more research is needed on the processes and outcomes [17]. Yet, the impact of the reform has resulted in more established PHCCs and an increased number of visits to primary care physicians (20%); but patient satisfaction is unchanged and professional opinions are mixed [17]. Half of all primary care visits in 2012 took place in private practices [8].

### The culture and climate for innovation

Many studies examining change processes in the public sector define innovation as intentional, or imposed and radical, stressing the existence of policy-directed pressure for change to which organizations adjust ([18]:70]). Greenhalgh et al. [19] show that a policy “push” can indeed increase early implementation of an innovation. Yet, the underlying approach to innovation has changed in recent years, with more emphasis on other driving forces for innovation [12]. Innovation may have its starting point in a “disruption” of practice [20], such as a new reform, but we cannot know what or who will be the future disruptors of health care. An alternative way to address the social challenges facing health care and to deliver a health care service for the twenty-first century is to empower the workforce with entrepreneurial skills [10], which places increased emphasis on performance improvement and systems for innovation grounded in daily practice [2, 12]. However, different organizations provide widely different settings for innovations. Studies of organizational process, environment, and culture explore an organization’s innovativeness, tending to concentrate on the prevailing culture and climate, notably in relation to leadership, power, relations, and attitudes [19, 21]. A receptive environment for change is characterized by “an absorptive capacity for new knowledge, strong leadership, clear strategic vision, good managerial relations, visionary staff in pivotal positions, a climate conducive to experimentation and risk taking, and effective data capture systems” ([19]:607]). Greenhalgh et al.’s literature review shows that initiatives to make the benefits of innovation more visible and understanding of improved task performance increase the likelihood of integration. Studies also show a strong link between innovation and leadership in the public sector [11, 22–25].

The key resource in many public services, such as primary care, is undoubtedly the staff’s expertise and capacity for problem solving [6, 21]. Staff’s internal sources of motivation, such as autonomy, expertise, and pro-social behaviour to safeguard patient needs, tend to have a greater impact on behaviour over time than external remuneration [26–28]. Intrinsic motivation can trigger a disinclination to be managed and controlled by compensation models that govern towards production, leaving less room for new thinking and acting on the basis of expertise and patient’s needs [29]. Thus, change must be accompanied by support structures and managerial actions, encouraging new behaviour and facilitating meaning making [12, 22, 30]. Innovations that are compatible with organizational or professional values, norms, and ways of working are more readily adopted [19]. With this approach, change processes tend to stress not only external drivers of change but also a more intrinsic motivation. Research has found that a learning-promoting climate and culture to mobilize human resources, a pro-innovation attitude in the organization, and establishing clear organizational goals that encourage achievement in innovative ways are important drivers of change [2, 12, 23, 28, 29, 31]. Arnaboldi et al. [6, 17] suggest that research needs to pay increased attention to the local conditions under which reforms and tools are applied to eliminate the negative side effects on the key resource of human capital and that “there is scope for exploratory research in study settings where successful performance managements appear to operate.” Also, Greenhalgh et al. ([19]:618]) highlight the need to understand the kind of culture and climate that supports and enables change. Thus, there is a need to take a broader view than simply managerial reforms and open the black box of how reforms and incentives are managed and integrated in daily work [32, 33]. This study was performed in relation to the introduction of a new reform in Sweden. We justify the inclusion of success cases to gain insight into the “black box” problem, as to how external demands are successfully managed.

The purpose of this study was to explore success cases to identify what enables primary care innovation. The question addressed is: What do the PHCCs have in common that enables primary care innovation? Supported by Osborne and Brown’s [34] definition of public sector innovation, we define innovation as the introduction of new elements into a public service, representing a discontinuity with the past.

## Methods

This study had a qualitative design and was based on 48 in-depth interviews with managers and staff at five PHCCs in the Jönköping region. The COREQ checklist for qualitative studies was used to assure quality standards [35].

We employed Success Case Method (SCM) because we believed it had potential for helping us to develop a better understanding of the mechanisms through which a new reform leads to envisaged change, given the context of change in a complex practice setting. SCM is often designed to identify and report on the apparent factors that made a difference between success and lack of it [36]. However, it has been used to learn from only success cases, with the advantages of easier access to busy professionals, greater willingness to participate with positive experiences, and an opportunity to analyse cases in greater depth [37]. In this case we looked for and documented the best cases in the Jönköping County Council, in relation to the introduction of the National Choice of Care Reform, and captured the essence of these positive experiences.

### Study setting

Swedish health care is financed by regional taxation, and payment is bound to the organization rather than to the individual physician. Payment systems vary among Swedish County Councils [8], suggesting that within a country’s broader reform, strategies are quite disparate [4]. Today, most PHCCs receive close to 90% of their income from fixed payments/capitation. In the Jönköping region, where the study took place, the National Choice of Care Reform was fully implemented in 2010. The income for the centres comes from three major sources: 82% fixed payments; 6% from quality imbursements; and 12% from special reimbursements, such as conducting health curves on predetermined target groups. Aside from income from the County Council, there is also a small fee for each visit. Also, the region has a long-standing culture of improvement work [38, 39]. At the time of the study, there were 46 primary care units, 31 public and 15 private, in the Jönköping region. Personnel at the regional unit responsible for primary care goals and follow-up systems assisted us in the selection of success case sites. To qualify as a success case, a PHCC needed (1) to provide high-quality care according to national and regional comparisons (e.g. national patient surveys); (2) have a low staff turnover; (3) positive financial development; (4) good leadership (in terms of living up to the reform goals, as entrepreneurs, or changing red figures for the better); and (5) high scores on the regional quality incentive scheme.

### Data collection

Five PHCCs were purposively selected to ensure inclusion of both public and private facilities, resulting in a sample of three large urban public units, two with smaller branches, one large urban private unit, with a small subsidiary, and one small rural private unit. The informed consent process began by contacting the five managers, providing information about the study design, research ethics, and requirements in terms of time for the participating staff. An informed consent letter was signed before the managers began to purposively recruit employees to their staffs. All but one professional agreed to participate. Data was collected through interviews. A questionnaire was sent to participants a week before the interview, including background information such as age, education, work experience, occupation, number of years, and role at the care unit. The process resulted in a sample of 48 participants aged 29–67 years (33 female, 15 male). Sixty-one percent were employed in connection with the introduction of the reform, and 39% were already employed. Seventy-two percent had 11 or more years of experience in health care. Of the five managers (2 female, 3 male), four had a nursing background and one was a physician. A summary of the study population’s and PHCCs’ characteristics is presented in Table 1.

**Table 1:** Sample distribution across professional groups and characteristics of the PHCCs

Units	A	B	C	D	E	Total
Listed patients	11522	14578	13154	3665	14221	57140
Public (Pu)/Private (Pr)	Pu	Pr	Pu	Pr	Pu	
Administrators	2	2	3	1	1	9
GPs	3	2	2	1	1	9
Nurses	3	4	3	1	2	13
Managers	1	1	1	1	1	5
Other staff members	1	2	1	4	4	12
Total	10	11	10	8	9	48

The interviews lasted for 35–65 min. All interviews were carried out at face-to-face meetings at the workplace from May to December 2015. A semi-structured interview guide was developed for this study and used to enhance dependability. The guide included five themes, which originated from what previous studies had reported to have a direct bearing on an organization’s innovativeness:

- Organizational culture
- Goals
- Change and improvement work
- Personal attributes
- Leadership

The interview guide is presented in Additional file 1.

All interviews were recorded and later transcribed verbatim. The transcripts were coded to ensure confidentiality. An informed consent process was initiated and verbal consent was obtained from participants, who were given assurances of confidentiality.

### Data analysis

The 48 interviews were analysed using a qualitative approach with content analysis to identify practice changes [40]. In the first step, all researchers read the transcripts in their entirety to gain a sense of the whole. Then, the first author marked and recorded interesting passages that responded to the study aim in a table, centre by centre (A to E). Initially, the focus was on identifying changes in practice. The changes were analysed and discussed with the research group, and then informed by commonly defined types of innovations [12, 21, 23, 41], categorized into three types of innovations: (1) services innovation, (2) process innovation, and (3) organizational innovation. The aim of the next reading was to identify data on practice features, and a tentative list of items was compiled by the first author and expanded by the others. The factors were discussed and grouped into meaningful categories by the research group.

## Results

The results identified practice changes, which were categorized in three types of innovations: (1) service innovation, (2) process innovation, and (3) organizational innovation (Table 2). The main features that enabled primary care innovations at the PHHCs were then delineated.

**Table 2:** Types of innovations

1. Service innovation	a. E-health services (booking services, e-physician) b. Altered opening hours c. Health-Enhancing Physical Activity (HEPA) d. Multi-professional teams to handle specific patient groups with joint needs (dementia, diabetes, rehabilitation).
Definition: New services that have been launched, which involve changes in the capabilities of services. Service inno - vation can be defined as delivering new services to existing users. This category also includes significant improvements	in e.g. technical specifications, software, user friendliness or other functional characteristics. Both entirely new services and improvements to existing services are included.
2. Process innovation	e. Triage, referring patients directly to paramedical personnel rather than to the physician f. Unit for ulcers
Definition: New or changed service processes, including how existing services may be provided to new users^a^.	
3. Organisational innovation	g. Drop-in units h. Integration of medical and healthcare centre i. Life insurance/Insurance medicine service j. Occupational health service k. Educational centre for international physicians
Definition: New ways of doing business. This includes the implementation of new organisational methods in business practices, workplace organisation or external relations. Also,	including innovations in structure, strategy and administrative processes.

^a^New users are new enlisted patients

### Service innovation

Services innovations are multifaceted. At first sight, new services launched at some of the PHCCs, such as e-health services (a), or altering opening hours (b), may seem “simple”, but these innovations, like all innovations, required new ways of working, and often involved coordinating different professions.

Teams involving various professions to handle specific patient groups with joint needs (dementia, diabetes, rehabilitation) were created at the PHCCs (d). One target of the reform was to administer the increasing need for rehabilitation. In Sweden, people on sick leave for more than 28 days are automatically summoned to health care, most often primary health care, for a rehabilitation plan. A rehabilitation team commonly includes a physiotherapist to assess the patient’s fitness, an occupational therapist to look at the home environment, a physician to undertake a physical examination, and a specialized nurse for conversation therapy. It is also common to have representatives from the municipality on the team. The ability of PHCCs to provide multimodal teams for patient groups with joint needs has greatly improved the service for patients who previously needed hospital care. Before a return visit to a physician becomes necessary, other professionals see the patient continually, which means more frequent contact, better care, and greater value for the patient.

### Process innovation

New or changed service processes were created when professionals were given new mandates for assessing conditions that previously belonged to physicians (e). In this way, managers enabled less expensive professionals to do progressively more sophisticated tasks.

The patient flow was reorganized at two of the PHCCs by allocating and prioritizing patients to the most suitable profession for an initial consultation, a so-called triage (e). If, for example, a patient had back pain, the patient was referred to a physiotherapist, who made a first assessment, treated the patient, and, if necessary, referred the patient for radiography, without having to consult a physician. Results showed that 95% of the patients were directed immediately to the most competent profession. This innovative solution resulted in money being “saved” because physiotherapists cost less than physicians, which in turn allowed for new recruitment.

A unit for handling ulcers was implemented at one of the PHCCs (f). The regional county considered the new process to be truly innovative and effective, and granted the PHCC an award. When starting the ulcer unit, the healing process for an ulcer was 25 weeks, which was above the national average. By reorganizing, providing further education, and allocating responsibility to nurses, this time decreased to 12 weeks. Then, new equipment was bought and the treatment process is now 4 weeks.

### Organizational innovation

Organizational innovation encompasses new ways of doing business, and the PHCCs created this by adding new business to their core activities (i, j, k), or by altering business methods (g, h).

For some PHCCs, doubling the number of new patient consultations in the last 3–4 years became a reality. Drop-in units (g) were one way to address the waiting lists for patients in need of care. An entirely new branch, offering only drop-in visits between 8 am and 4 pm, 5 days a week, was started at one of the PHCCs. At the drop-in unit, no scheduled meetings for the staff or patient bookings were approved. However, lunch breaks were occasionally used for staff meetings, although on paid time including a free lunch. An alternative drop-in organization was set up at another PHCC, offering patients doctor’s consultations each morning preceded by an open phone line (for 1 h) to the nurse’s office.

Collaboration with new parties, such as a fitness centre, the Jönköping International School of Business, and companies offering life insurance, was another way to innovate practice (i). One of the PHCCs had invested in a centre integrating health and well-being (h). The fundamental idea behind the newly opened centre was that more proactive, preventive care would lessen patients’ need for medical treatment. It is recognized that lifestyle-related health problems, such as inadequate physical activity, unhealthy diet, and tobacco use, are increasing in Sweden, which results in extensive costs to society. To realize the vision, the PHCC used capitation-linked payment to recruit a quite different staff setting, e.g. nurse assistants working with health care prevention and specialized nurses offering conversation therapy. Also, an agreement was made with a fitness centre to run a gym on the premises, allowing the patients highly accessible and functional workout practice. Cost effectiveness was reached by offering group activities, such as activities for high-performance achievers who lacked an active circle of friends, thus constituting a potential risk of developing lifestyle-related health problems.

One PHCC had developed an educational centre for international physicians to convert their license to Swedish standards (k). Supervisors were educated to care for and instructed to lead the foreign doctors in clinical settings. The manager acknowledged that the educational centre was the most important effect of the reform, because the risk of safety and quality issues that followed too many shifts in this group of physicians was minimized, as was the need for short-term locum physicians.

### Features of the practices

The analysis yielded four main features of the practices: (1) managing learning, (2) monitoring performance, (3) adapting to requirements, and (4) collaborating with others. The citations are coded by profession and health care centre (Table 3).

**Table 3:** Main features and examples from the interviews

	Quotes from the interviews
1. Managing learning	
• The leader strived to provide good conditions for creativity, and readiness to encourage experimentation.	*If I know that a patient is seeing a physician for varicose veins, I don’t hesitate to knock on the door after the examination, and ask the patient if I could examine her veins. That is how you learn. It is all about practicing. (Nurse assistant E)*
• The leaders organized their accessibility, such as allocating consultation time for staff members´ questions during the workday.	*The manager has great faith in us and says that I am more qualified to handle certain patients than a physician. (Occupational therapist E)*
• High demands were made on the staff to take professional responsibility and contribute to quality health care services.	*I have my own patients 2 days a week […] the doctor refers patients with minor hypertension to me, and I’m responsible for summoning patients for blood pressure checks and consultations about their lifestyle, diet, and physical activity. (Nurse assistant A)*
• Time was provided to handle problems and hurdles in ordinary work, and fulfil the county council’s requirements of reporting two improvements per year.	*By offering proactive health activities, we teach patients more about how they function and contribute to public health. (Manager D)*
• The leaders were highly goal-oriented and communicative about their vision.	*We will not pursuit profit in the welfare sector; we will reduce costs. (Manager B)*
• The leaders’ appreciation for staff engagement was reinforced in different activities, such as arranging summer parties for the staff and families, monthly social coffee breaks together with the children, or an annual salary bonus.	*Our vision is to be the best primary care unit at providing good health care. (Care administrator A)*
• A recurrent activity was to use lunch breaks, morning meetings or afternoon coffee breaks as educational settings. This was also a way to deepen the staffs’ awareness of conditions in the reform, such as performances that contribute to increased goal achievement.	*It (triage) is somewhat of a success […] we save time, suffering, and money […] triage has affected other professional groups, a true professional development for many, which has enhanced their legitimacy. The effect is inspiring, making other professional’s worth visible in the care process by handling tasks that used to belong to the physician. (Manager E)*
	*Although lunch is paid for, having too many lunch meetings in one week becomes stressful. (District nurse B)*
2. Monitoring performance	
• Many of the tools afforded at the workplace functioned as understandable and valuable feedback mechanisms, but were not always reassuring.	*The manager involves us in economic issues, which leads both to participation and shared responsibility (Care administrator C)*
• Performance measurements were given in different settings (staff meetings, morning gatherings, white board, weekly newsletters), and visually (tables, figures, colours).	*It is important that we make our goals transparent and can follow progress […] we become more aware and can develop better services. But, there is more focus on finance now, everything has a price […] these kinds of incentives tend to start a surfeit of diagnoses and develop a system that can be manipulated. (Nurse E)*
• Some tools for monitoring services were introduced parallel to the introduction of the reform, such as the regional county´s endorsement of a shared electronic medical record system with the hospitals in the region. Other tools gradually developed professional autonomy.	*People are aware in a completely new way. They can change health care units, and make demands, as consumers. (Care administrator E)*
	*It is stressful to every day see the aggregated sum of dictates that need attention on my computer screen […] but, at the same time, I am triggered when our efforts align with our own goal of transcribing the dictates within 48 hours. (Care administrator C)*
	*patients in a more efficient way. (Physiotherapist D)*
3. Adapting to requirements	
• The reform created disturbances in practice, and triggered a need to change routines.	*The increasing administration has forced us to examine our routines to relieve pressure on the doctors. The nurses have taken over some of their work, which in turn has led to new tasks for us. There are many advantages to this rearrangement, more time for patients and professional development for us. (Care administrator C)*
• Adaption to compensation rules was necessary for survival, but also needed to realize visions and create patient value.	*Financial incentives do not destroy professionalism and autonomy, because concerns about monetary resource are included in all sound public finances […] we perform and report as requested, and can use the money creatively. (Manager B)*
• Many staff members stressed that health care must be available to all citizens, because taxation is the basis of their existence.	*I’m not incited by them, but by the management’s desire to provide good care. (Physician B)*
• Managers overruled the financial logic of the reform to safeguard patient needs and reinforce professional values.	*Actually, today we have to perform something to get paid. (Rehabilitation coordinator C)*
• Financial incentives were translated to attract professional values	*We do not talk about financial goals, rather that we have certain areas that need special attention. (Physiotherapist E)*
• Not all incentives were appreciated and created frustration when regulations circumscribed possibilities to be innovative.	*We are here to provide care, to help. If a person seeks help, the first thing we ask is ‘Are you listed here (at this PCU)?’. (Nurse assistant C)*
	*We have decreased our development rate because we are so busy producing. (Occupational Therapist E)*
	*I do not have the authority to relocate the organization for monetary and work environmental benefits. (Manager E)*
4. Collaborating with others	
• Interaction among various professionals in a team allowed for a more holistic approach and was believed to provide better continuity for the patients.	*We used to be divided by hierarchy, nurses and nurse assistants, there was no understanding for what we did, but that has changed completely […] today we work together, side by side. (Nurse assistant C)*
• The staff experienced that new ways of doing work reduced the administrative workload, supported teamwork and attracted new patients.	*With the drop-in unit, we quickly respond to the patient in need, not several weeks later […] no scheduling or rebooking is necessary. (Nurse B)*
• Crossing of professional boundaries was evident in practice.	*We solve problems together. You never feel completely alone with something, so if there is a problem, the work climate is so unpretentious that anyone can ask anybody. (Physician A)*
	*Multimodal rehabilitation requires two visits a week for 8 weeks, with at least two professions in addition to the physician […] my role is to monitor the process and inform the physicians of the patients on registered sick leave, but also the care administrator has a role in the goal fulfilment. This is true teamwork. (Rehabilitation coordinator C)*
	*I don’t think there is any physician here who wouldn’t fetch me if they have a patient with an ulcer…they say to the patient that I am an expert. (Nurse assistant E)*

## Discussion

This study identified three kinds of innovations, which break with previous ways of organizing work at the PHCCs: (1) service innovations, new services that have been launched, which involve changes in the capabilities of services; (2) process innovations, new or changed service processes; (3) organizational innovations, new ways of doing business. Although the innovations are categorized into three types, the requirement to meet multiple goals and increasing complexity in an organization often demand innovativeness across a range of innovation types [21, 41].

The following characteristics were common to all the PHCCs: the patient flow had increased, doubled in some cases, for new patient consultations in the last 3–4 years; the leadership was highly appreciated; inter-professional interaction was encouraged; increased evaluation activity was experienced; and a long-standing culture of improvement work was valued. There was increased awareness and responsibility towards work performance and goal clarity. Conscious deliberation of performance and goal achievement became a natural aspect of daily practice for many professionals. It is reasonable to assume that innovation was possible due to a culture and climate of *managing learning*. The fertile ground for innovativeness was supported by the design of the reform in this County Council. That is, market forces, such as competition and openness, pooled with a relatively extensive system of regulations, joint information systems and quality improvement activities, allowed the PHCCs to *monitor performance*, *adapt to requirements* and *collaborate with others* to support primary care innovation.

### Comparison with the existing literature

It is widely recognized that primary care is subjected to political influences and is repeatedly reformed [1], suggesting that service innovation is a necessary ingredient for success [12]. The two reviews on the reform show differences in the way strategies were designed, conceived and implemented across Sweden [8, 17]. In the Jönköping region, we found a favourable mix of market forces related to the “Nordic Model” for health care systems, such as competition, openness, and innovation in combination with public regulations, to eliminate unreasonable profits and corruption [4]. In Sweden, as the reform moved from PHCC being responsible for a geographic population to freedom of choice for the patient, the PHCCs in this study increased patient numbers by offering innovative solutions, such as decreasing the waiting list with drop-in units, handling rehabilitation in multimodal teams and developing new alliances with other parties, at the same time as they attracted staff values of social justice, i.e. providing quality health care for all citizens. One of the underlying tenets of professionalism is the commitment to the patient’s needs and to justify work and decisions in relation to these needs. As shown elsewhere [23], managers overruled the financial logic of the reform to safeguard patient needs and reinforce professional values. For example, at some PHCCs, the manager decided not to complete health curves, because they were not considered useful for creating good health care services; rather they were believed to exploit valuable time, even if they could contribute to “easy” financial revenue. A key performance measure in recent years has been the use of waiting lists for patients in need of health care treatment [6]. The two new drop-in units radically reduced the waiting lists and put the PHCCs on the regional top 10 score list. However, a concern among many professionals was the understanding that the most demanding patients with intricate problems, involving multiple body systems, still suffer despite the reform, because they are too weak to navigate the changing system. The units reinforced professional values because they enabled a quick response to patients in need, but at the same time were discouraging with regard to patients not fit enough to handle the changing system.

Change in practice is influenced in many more ways than politics. First, we suggest that the long tradition, support and familiarity of improvement work in Jönköping County Council provided fertile ground for innovation [27, 38]. Second, we believe that the mix of professionals in Swedish primary health care has great potential for the emergence of new practices. The interplay across professional fields and between professional knowledge bases disturbed routines and tended to form opportunities for new thinking and acting. The changing professional boundaries had implications for professional fields and responsibilities, and by cross-boundary collaboration, it was possible to use and share the knowledge base of different professions in creative ways [28, 42]. Professional gaps and organizational spaces that affect patients negatively could be avoided by increased collaboration. Easy access to digital support systems shortened the communication channels across professions. Third, as shown in previous research [11, 22–25], leadership was an important factor to create a culture and climate that encouraged experimentation. To different degrees, all leaders had entrepreneurial characteristics, such as being visionary, having a pro-innovation attitude, believing in competition and showing immense engagement in improvement work [11, 19]. The leaders in our study had the ability to develop the workplace as a learning environment and utilize performance measurements such as evaluations and process indicators to involve the staff in performance outcome [2, 12, 31]. In our study, we also found that the leaders had the ability to govern the content of the reform towards targeting professional values and intrinsic motivation. Fourth, the impact of management on organizational performance is suggested to be dependent on characteristics of the workplace [11, 19, 41]. For example, is feedback an essential aspect of a deliberative practice [42]. As shown in our findings, accessible feedback tools were applied and also developed autonomously at the PHCCs, thus reflecting a professional stance not just a leadership issue. To be professional calls for an awareness and reflexivity of both oneself and one’s work; the professional worker solves problems through creatively reflecting in complex service realities [42]. In our study, the importance of the professionals’ awareness and involvement in creating sustainable change is depicted, as well as their use of embeddedness in work practice as a positive basis for implementing desired change, which is supported in previous studies [2, 42].

### Strengths and limitations

As in all qualitative research, this study is limited with regard to generalizability, especially as the design of the reform varies across Sweden. Transferability is however enhanced by the variety of PHCCs included. The study was conducted at PHCCs assessed as success cases and in a setting with a long tradition and support for improvement work. The concept of success is complex and the research group is aware that the description used is inadequate in capturing such a complex concept. In addition, organizations are in continuous movement; during the time between the acceptance and planning of the study and the data collection, it is an interesting observation that three of the five PHHCs are the only units in the region with the same managers since the reform in 2010. Thus, discussing sustainability when referring to an organization being successful is also important. Although it was our ambition to provide a better understanding of how reforms and incentives are managed and integrated in daily work, the purposive sampling may have affected the results. However, as previous studies have reported many negative responses related to reforms, the risk of social desirability is likely less perceptible. With regards to the managers’ involvement in the sampling process, the risk is lessened by the large sample from each PHHC. Also, the large multi-professional interview sample, with various professional backgrounds, strengthens the credibility of the results and creates a more comprehensive picture of change in work. However, a limitation is that we did not compare successes and non-success or attempt to explain variations in the degrees of success between cases. Neither were the results analysed across professions. Yet, a strength was that the findings have been tested and verified by the professionals during return visits to the PHCCs. Additional studies exploring the links between reforms and practice-based innovations at PHHCs not defined as success cases would be of interest for further research.

## Conclusions

This qualitative study highlights critical features in practice that support primary care innovation. A learning-oriented culture and climate, comprising entrepreneurial leadership, cross-boundary collaboration, visible and understandable performance measurements and ability to adapt, are shown to be merits for innovativeness. The manager’s ability to transform and integrate a policy “push” with professionals’ understanding and values can better support primary care innovation. Ultimately, the key to innovation is the professionals’ willingness, capability, and opportunity to innovate.

## Additional file

Additional file 1: Interview guide: Interview questions, including supplementary questions to the managers. (DOCX 86 kb)

## Abbreviation

PHCC: Primary health care centre
