# Supplementary material for: Tending to innovate in Swedish primary health care: a qualitative study
Source: BMC Health Serv Res. 2019 Jan 18;19:42. doi: 10.1186/s12913-019-3874-y (PMC6339427; doi:10.1186/s12913-019-3874-y)
Supplement: Supplementary file 1 — Interview guide: Interview questions, including supplementary questions to the managers. (DOCX 86 kb) [file 12913_2019_3874_MOESM1_ESM.docx]

## Interview guide AFA

20 April 2015

## Questions to the manager at the PHCC

How is this health care centre managed?

Who is held accountable for the unit in regards to quality, financial, personnel and work environment issues?

Who are the responsible managers? Do you have a steering committee?

How do you develop a genuinely good health care unit?

How would you describe the work culture and climate at this unit?

**________________________________________________________________**

**Opening question:** What role do you have at the health care centre?

## Organizational culture

Have you made an active choice to work at a (private/public) health care centre?

Have you worked in other forms of ownership?

How would you describe your workplace environment?

What characterizes a well-functioning workplace?

What do you like/dislike about your workplace?

## Goals

How has the reform and its requirements affected your work?

Are you aware of your organization’s overall goals? If so, how do these objectives apply to your work?

Are there any targets that are attached to your work especially?

How do you keep up with goal achievements (overall and for your own work)?

What support do you have to reach declared objectives?

Do you feel involved in formulating and reaching these objectives?

What makes you willing to reach the objectives?

**Consequences:** Do you see any consequences of working more goal oriented (for the patients, as a professional, for the unit)?

## Change and improvement work

How would you define change in your line of work?

How would you describe your organization’s interest in improvement work?

Is there a support structure for improvement work?

What do you do when you have a new idea that you believe will improve some aspect of your work?

Can you tell us about a change of work that you have been involved in?

## Personal attributes

What motivates you to engage in work?

In what way does the declared objectives effect your willingness to engage in work?

What drives you to create something other than traditionally done in care work? Can you give an example of something you have succeeded with at work?

What has occurred on a day when you get off work feeling particularly happy?

Can you provide examples of ethical dilemmas in your work? Do you have experience of you or your colleagues doing things that possibly gain the unit more than the patients?

Are there situations when you go beyond that of providing necessary requirements for the patients? Can you give an example?

## Leadership

What characterizes good leadership? What do you expect of your manager?

What does your manager do that you (particularly) like?

How would you characterize your manager’s leadership?

What does your manager do to motivate you to engage in work?

In what situations does your manager/the management talk about the unit’s objectives?

How does the manager follow-up on the objectives?

Do you find support in issues that are stressing in your work?

**Question to the manager:** What is important for you to acknowledge in your leadership?
